# Design and Implementation of Graphene-Based Tunable Microwave Filter for THz Applications

**DOI:** 10.3390/nano12244443

**Published:** 2022-12-14

**Authors:** Cleophas D. K. Mutepfe, Viranjay M. Srivastava

**Affiliations:** Department of Electronic Engineering, Howard College, University of KwaZulu-Natal, Durban 4041, South Africa

**Keywords:** graphene, silicon, silicon dioxide, substrate-integrated waveguide, chemical potential, nanotechnology, nanomaterial

## Abstract

A reconfigurable Substrate-Integrated Waveguide (SIW) filter operating in the THz region was designed in this work. Two SIW resonators were coupled through a magnetic iris to form a second-order filter with a double-layer substrate. The first substrate was silicon of permittivity 11.9; on top of it, silicon dioxide of permittivity 3.9 was placed. The ground and upper plane were composed of gold plates. Graphene material was then used for the tunability of the filter. A thin graphene sheet was sandwiched between the silicon dioxide substrate and the upper gold plate. An external DC bias voltage was then applied to change the chemical potential of graphene, which, in turn, managed to change the operational center frequency of the filter within the range of 1.289 THz to 1.297 THz, which translated to a bandwidth range of 8 GHz. The second part of this work centered on changing the aspect ratio of the graphene patch to change the center frequency. It was observed that the frequency changed within the range of 1.2908 THz to 1.2929 THz, which gave a bandwidth of 2.1 GHz change.

## 1. Introduction

Microwave filters are a fundamental component of communication systems. With recent technological advances, tunable/reconfigurable filters are becoming useful to achieve the requirements of modern filters. Beyond 5G, communication systems are required to operate at high frequencies in the region of THz. Various filters use planar technologies, which suffer considerable losses in the THz region, including various materials [1,2,3,4,5,6,7,8,9,10]. Substrate-Integrated Waveguide (SIW) filters were introduced to solve the problem of high losses at high frequencies suffered by planar circuits. The SIW has the same characteristics of high-power handling and lowers losses at high frequencies as the conventional waveguide [1,2,3,4].

In terms of tuning the filters, different methods have been explored in the literature [5,6,7,8,9,10,11,12]. In [5], a liquid metal (GaInSn) was used to tune the center frequency. The volume of the cavity was reduced by filling the cavity with this alloy, and in turn, this reduced the internal wall distance. The frequency tuning was between 7 GHz and 8 GHz. In [6], a multi-layer periodic structure was presented whereby tuning was achieved by liquid metal. A thickness-controlling aperture separated the Frequency Selective Surface (FSS). The liquid metal layers were combined with the elastomeric substrate to produce a flexible and fluid-tunable device. The filter was tuned between 13.5 GHz and 17 GHz. Alkaraki et al. [7] presented a method for allowing or preventing wave propagation with a removable wall built from several drill holes. To form a wall, the vials were filled with liquid gallium metal, and when not required, the vials were emptied. Tuning occurred from 4.7 GHz to 7.2 GHz

Qian [8] used a liquid metal, Eutectic Gallium Indium (EGaIn), to design a reconfigurable meta-surface antenna. A distinct reflective phase response was achieved by melting and rotating the element structure, which changed the liquid metal’s shape and achieved reconfigurability. Prasetiadi et al. [9] presented a liquid-based tuned filter. Square substrate-integrated waveguide resonators were capacitively loaded with liquid metal posts, which meant changing the loading capacitance. This technique changed the resonant frequencies between 3.4 GHz and 5.83 GHz. Sirci et al. [10] used varactor diodes in a back-to-back setup to tune the center frequency and bandwidth of the filter, as well as the return loss. Ten varactor diodes were used to implement a two-pole tunable band-pass filter with two transmission zeros. The tuning occurred in the range of 5.65 GHz to 6.35 GHz.

Li and Yang [11] placed varactor diodes along the open side of a half-mode SIW and microstrip feeding line to construct in-band notches. They then controlled the biasing voltage and reconfigurability was achieved. The frequency and bandwidth of the filter were successfully tuned independently. Frequency tuning was achieved in the range of 5.99 GHz to 10.15 GHz. Pal et al. [12] presented a stub-loaded resonator. A varactor diode was placed in the ring to control the odd-mode resonant frequency. The frequency was tuned from 0.69 GHz to 0.88 GHz, with a maximum insertion loss of 1.83 dB.

For high-frequency structures in the THz region, graphene has been used for frequency and bandwidth tuning [13,14,15,16,17,18]. This is achievable because graphene, as a material, has superior structural, mechanical, and electrical properties [19]. Ali et al. [13] used graphene for the tunability of a THz duplexing MIMO antenna. The antenna can tune the frequency at the two ports independently. By changing the graphene’s chemical potential, the radiators can be tuned. Kiani et al. [14] studied square patch microstrip antennas. Graphene material was used to control and regulate the antenna’s polarization. By simply controlling the fermi energy level, right-hand and left-hand circular polarization, and linear polarization can be displayed. This changes the chemical potential of graphene. Ram et al. [15] simulated a planar THz filter designed based on graphene. A graphene layer was inserted between the conductor layer and the dielectric to support the propagation of plasmonic waves. The graphene’s chemical potential was varied to tune the resonant frequency of the band stop filter over the range of 0.1 THz. Naghizade and Saghaei [16] presented a Graphene-On Insulator (GOI) band stop filter operating in the mid-infrared region. To achieve the tunability of the filter, some physical parameters, such as the number of overlapped filters and the number of graphene layers, were changed together with the applied voltage.

Moazami et al. [17] presented a plasmonic tunable single-stub filter. A thin graphene layer was used to tune the filter in the THz region. By applying a gate voltage between the gate graphene sheet and the indium antimonide (InSb) substrate, the center frequency was shifted by 32 GHz. The filter’s bandwidth was improved by 33% by increasing the number of stubs. Varshney et al. [18] presented a single/dual-band notch filter for THz applications. A graphene patch was inserted in the filter for tunability. The aspect ratio of the graphene patch was changed to determine the different TM_mn_ modes to be excited from the structure, which helped the filter to be configured as a single- or dual-band notch filter. The variation in the chemical potential of graphene also changed the center frequency. 

Graphene has the property of negative permittivity, and such materials are vital for the propagation of waves in the terahertz region. Negative permittivity is important in confining the propagation of surface plasmonic waves, which are waves that are transmitted on the surface of a metal and dielectric interface. Graphene is a two-dimensional (2D) material with semi-metallic properties. It is a single layer of graphite crystals with a single atomic layer of covalently bonded hexagonal carbon atoms. Graphene is composed of carbon atoms arranged hexagonally [20].

Graphene can support Surface Plasmon Polariton (SPP), which makes it a good candidate for electrical tunability. SPP is an electromagnetic excitation that occurs on the interface between a dielectric material and a metal surface [21]. Surface plasmons have very important characteristics, which include field localization, enhancement of subwavelength confinement, and high surface sensitivity. These properties give rise to various applications in the bio-sensing field and also integrated optical circuits. In [22,23,24], graphene was used in several sensing applications. In [22], a multi-mode surface plasmon resonance absorber based on a dart-type graphene single layer was presented. This was able to achieve tunability by adjusting the chemical potential and relaxation time of the graphene material. In [23], a one-step approach to fabricating magnetic Graphene Oxide (GO)/Poly (Vinyl Alcohol) PVA composite gels was presented. This was used to solve the problem of separating graphene oxide from water after adsorption. In [24], an ultra-narrowband graphene negative refractive index sensor was presented. The sensor achieved efficiency absorption rates of 99.41% and 99.22% at 5.66 THz and 8.062 THz, respectively. Tuning was achieved by adjusting the Fermi energy level and relaxation time. Graphene is tremendously good in electrical tunability and has low ohmic losses [25]. This is made possible through the change in the chemical potential of graphene, which can be achieved by doping or simply applying an external DC voltage. Using these electrical properties together with the optical properties of graphene, various components have been developed, including phase shifters, antennas, modulators, etc. [16,19,26,27,28,29].

In this research work, a second-order filter was built, which operates in the THz region. SIW technology was used. Two substrates were employed; the first was attached to the ground plate of silicon (SI) and the second was attached to the upper plate of silicon dioxide (SiO_2_). This filter can be used in bio-medical devices, such as sensors operating in the terahertz region. It can be used to let the required signals pass and forbid all other signals outside of the target frequency. This paper is organized as follows. Section 2 presents the graphene tunability for the microwave filter. Section 3 designs a tunable graphene filter. Section 4 discusses the results and the filter’s suitability. Section 5 presents the effect of the graphene aspect ratio on this filter design. Finally, Section 6 concludes the work and recommends future work.

## 2. Basics of Graphene Tunability

The tunability of graphene occurs due to the configurability of the surface charge conductivity defined by Kubo’s model [18,30]. The surface conductivity, $\sigma_{g}$, is a function of the chemical potential, $\mu_{c}$, which is a function of the applied external electrostatic field, *E*_0_. Surface conductivity is due to the contribution of inter- and intraband transitions through which the real part of the interband conductivity is negligible in the THz region of operation. This leaves only the intraband contributing to surface conductivity. The intraband conductivity can be represented as [18]:

(1) $$\sigma_{g1}=-j\frac{e^{2}k_{b}T}{\pi {ћ}^{2}\left(w-j2T\right)}\left(\frac{\mu_{c}}{k_{b}T}+2In\;\left(e^{-\frac{\mu_{c}}{k_{b}T}}+1\right)\right)$$

where *w* is the angular frequency, $\mu_{c}$ is the chemical potential, *T* is the temperature, *e* is the electron charge, ћ is the reduced Planck’s constant, and $k_{b}$ is Boltzmann’s constant. The interband conductivity can be investigated using:

(2) $$\sigma_{g2}=\frac{-je^{2}}{4\pi ћ}\;In\;\left(\frac{2\left|\mu_{c}\right|-\left(w-jГ\right)ћ}{2\left|\mu_{c}\right|+\left(w-jГ\right)ћ}\right)$$

where Г is the scattering rate. At lower frequencies, such as 0.5 THz to 5.0 THz, the inter-band conductivity of graphene remains negligible and the surface conductivity is only due to intraband transitions. However, the $\sigma_{g2}$ term at higher frequencies becomes complex with a negative imaginary part. Graphene material can be characterized by the displacement vector $D_{n}=\varepsilon_{b}E_{0}=en_{s}/2$, where *n_s_* is graphene’s two-sided surface charge density and $\varepsilon_{b}$ is the dielectric permittivity.

(3) $$n_{s}=\frac{2}{\pi {ћ}^{2}v_{f}^{2}}\overset{\infty }{\underset{0}{\int }}E\left(f_{d}\left(E\right)-f_{d}\left(E+2\mu_{c}\right)\;\right)dE$$

where $f_{d}\left(E\right)$ is the fermi–Dirac function as a function of energy, *v_f_* is the fermi–velocity ($\sim 10^{8}\;\mathrm{cm}/s$), and

(4) $$f_{d}\left(E\right)={\left(e^{\frac{E-\mu_{c}}{k_{b}T}}+1\right)}^{-1}$$

The tuning of the filter can be achieved by changing the voltage across the graphene sheet. This provides an easier alternative method for changing the central frequency of the filter than changing the entire geometry of the filter. The geometry of the filter can be changed in case there is a need to operate in a dual-band mode. The relationship between the surface conductivity and chemical potential is given as [31]:

(5) $$\mu_{c}={ћv}_{f}\sqrt{\frac{\pi \varepsilon_{r}\varepsilon_{0}V_{b}}{et}}$$

where *V_b_* is the bias voltage and *t* is the substrate thickness. The relationship in Equation (5) shows that, as the external bias voltage changes, the chemical potential also changes; hence, we can control the chemical potential of graphene using an external bias voltage, which, in turn, can be used to vary the center frequency.

## 3. Graphene Tunable Filter Design

**Figure 1:**
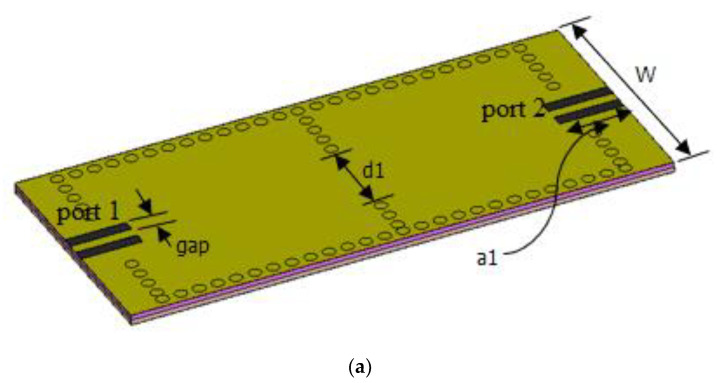

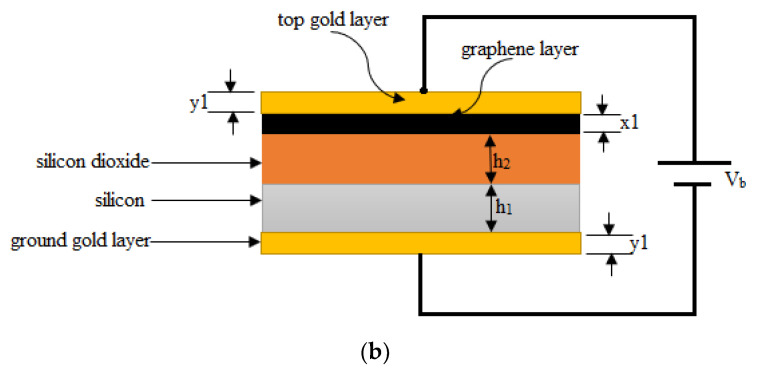
(**a**) Second-order SIW filter 3D image; (**b**) cross-sectional view.

Figure 1a shows a 3D image of the designed second-order SIW filter. The dimensions of the filter were arrived at by following the procedure in [2,32], where it can be seen that the width of the structure is proportional to the center frequency; hence, by changing W, the width of the resonator, the operational center frequency of the filter can be changed. The magnetic iris of length d1, according to the procedure in [2], provides the coupling between the two resonators. The external quality factor can be determined by varying distance a1 and gap, as shown in Figure 1b.

A band-pass filter was designed for operation at a resonant frequency of 1.2 THz. Figure 1b is the cross-sectional side view of the proposed filter. The top and ground plane were made up of gold of thickness 0.17 µm. A double layer of silicon (Si) and silicon dioxide (Si0_2_), with permittivity of 11.9 and 3.9, respectively, was also included. Graphene of thickness 0.0034 µm was placed between the Si0_2_ substrate and the top gold plate. The applied external DC bias voltage is indicated as V_b_.

**Figure 2:**
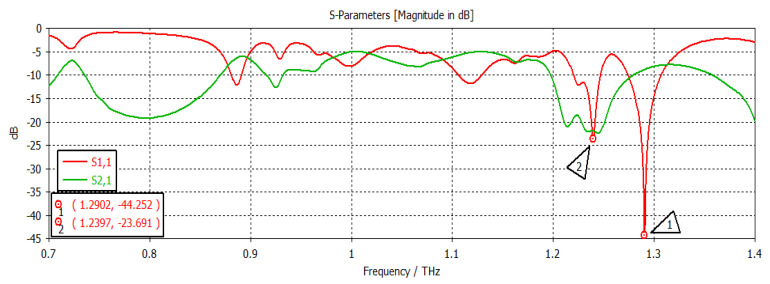
S-parameter results.

**Figure 3:**
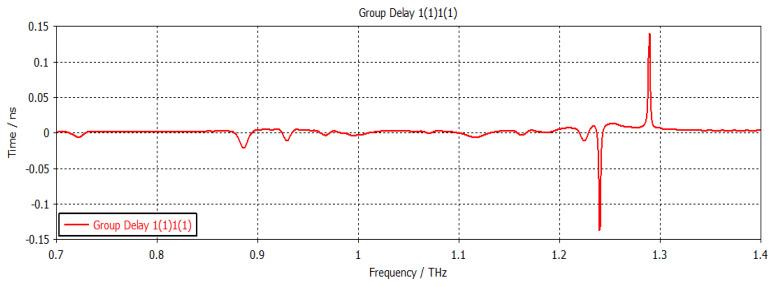
Group delay.

**Figure 4:**
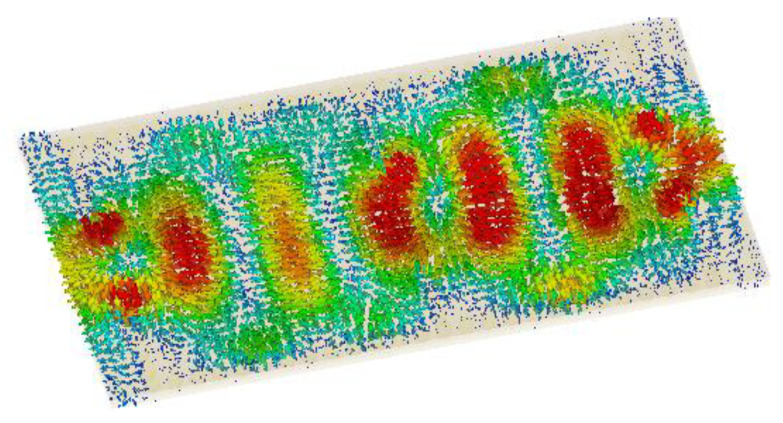
Current distribution.

**Figure 5:**
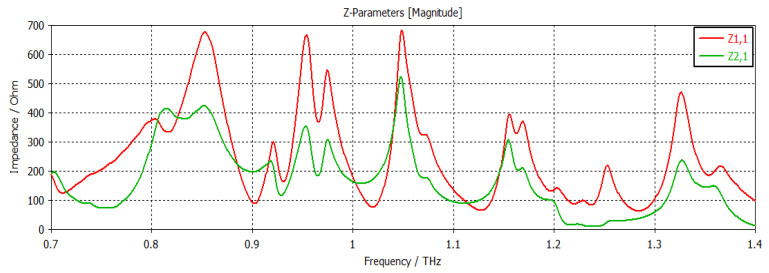
Impedance plot.

The structure was simulated at first with no graphene, as shown in Figure 2. This graph shows the return loss and insertion loss of the filter device. The SIW filter parameters are given in Table 1. It can be seen that the filter operated at a frequency of 1.2902 THz with a return loss of −44 dB. The group delay is shown in Figure 3. It can be seen that a group delay of less than 1.15 ns occurred at the edge of the passband. From the results, we can see that the group delay was very small, which is desirable; otherwise, a higher group delay will distort the waveform of the signals, resulting in unreliable communication. The surface current of the filter is shown in Figure 4. With the current being directly proportional to the electric field, this confirmed the electric field variations on the graphene patch, showing that the fundamental mode was being passed from port 1 to port 2 with a strong electric field on the surface of the graphene patch. Figure 5 shows the impedance plot of the filter, which confirms the resonance of several modes in the structure. The modes were quite close to each other and they could bring about a wider bandwidth band-pass filter if they could be merged together.

## 4. Results and Analysis of the Design

**Figure 6:**
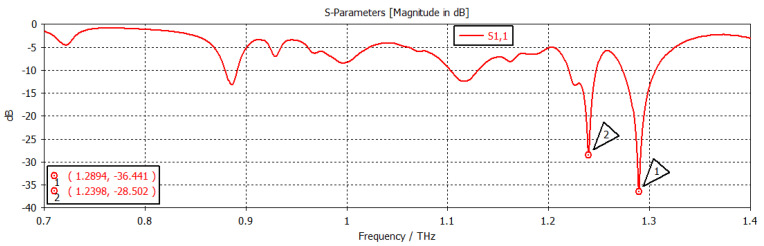
Chemical potential = 0.1 eV.

**Figure 7:**
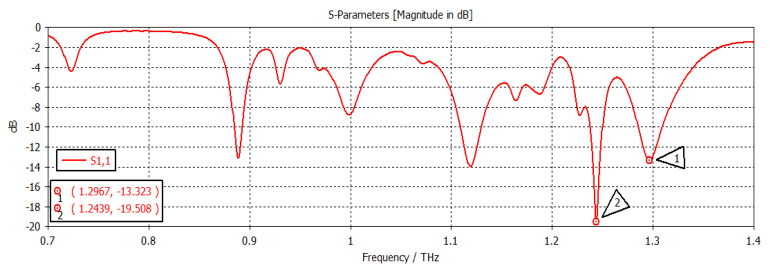
Chemical potential = 0.2.

**Figure 8:**
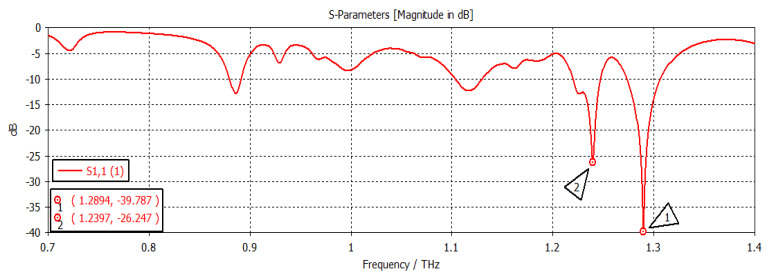
Chemical potential = 0.4 eV.

**Figure 9:**
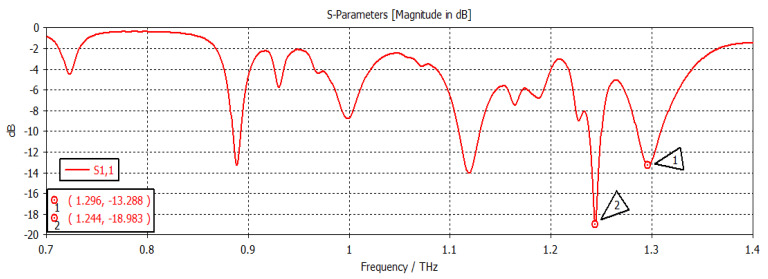
Chemical potential = 0.6.

In this work, the external voltage bias V_b_ was used to change the chemical potential of graphene. The values of chemical potential started from 0.04 eV to 0.40 eV. The other parameters of graphene are shown in Table 2. The chemical potential change was used to change the filter’s center frequency, as shown in Figure 6, Figure 7, Figure 8 and Figure 9.

When the chemical potential was 0.1 eV, the SIW filter resonated at first and second frequencies of 1.2894 THz and 1.2398 THz, respectively, with return losses of −36.441 dB and −28.502 dB, respectively, as shown in Figure 6. When the chemical potential changed to 0.2 eV, as shown in Figure 7, the first and second frequencies were 1.2967 THz and 1.2439 THz, with return losses of −13.323 dB and −19.508 dB, respectively. In Figure 8, the chemical potential changed to 0.4 eV and the results show that the first and second frequencies were 1.2894 THz and 1.2397 THz, respectively, while the return losses were −39.787 dB and −26.247 dB, respectively. Finally, the chemical potential was set at 0.6 eV, as shown in Figure 9. For this instance, the first and second frequencies were 1.296 THz and 1.244 THz, respectively, with return losses of −13.288 dB and −18.983 dB, respectively.

As shown in Equation (5), the chemical potential of graphene is directly proportional to the applied bias V_b_; hence the external voltage was varied to obtain different chemical potential values, and in turn, the center frequency changed.

## 5. Effect of Graphene Aspect Ratio on the Center Frequency

**Figure 10:**
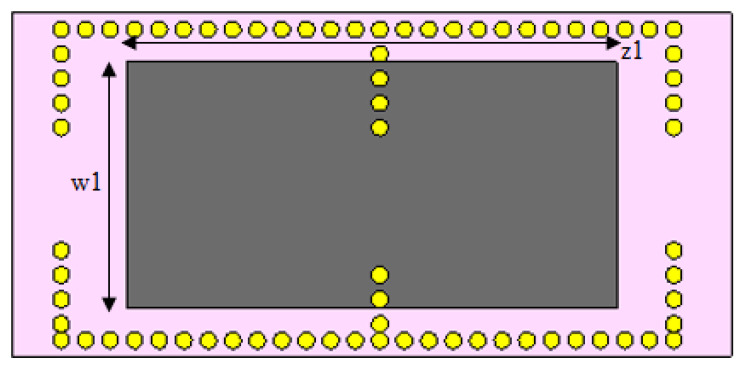
Length and width of graphene.

The length and width of graphene, as shown in Figure 10, were changed simultaneously, and the effect on the center frequency is presented here. The top layer of copper, as shown in Figure 1a, was removed to expose the graphene sheet, shown in Figure 10 as the grey patch with dimensions w_1_ and z_1_. Here, yellow circles represent the via holes.

**Figure 11:**
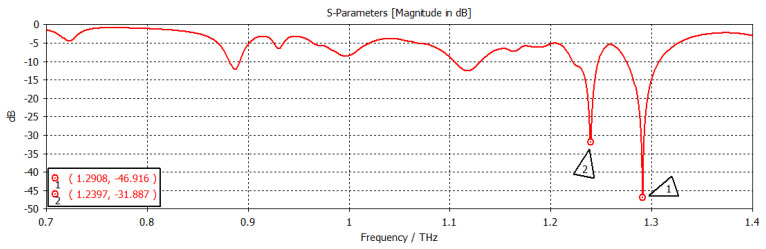
w_1_ = 50 µm and z_1_ = 100 µm.

**Figure 12:**
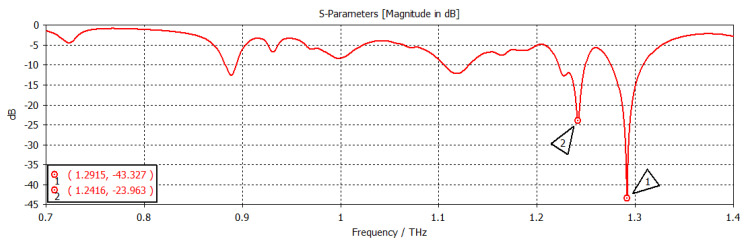
w_1_ = 50 µm and z_1_ = 300 µm.

**Figure 13:**
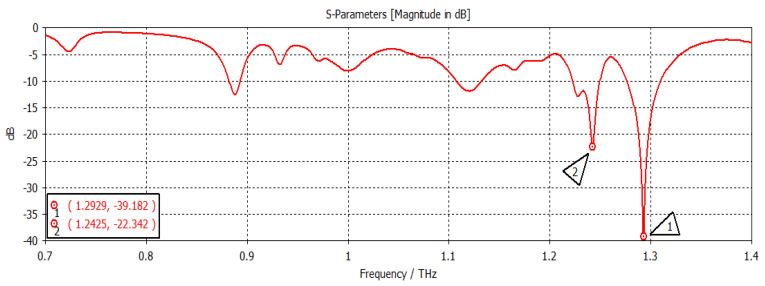
w_1_ = 100 µm and z_1_ = 300 µm.

**Figure 14:**
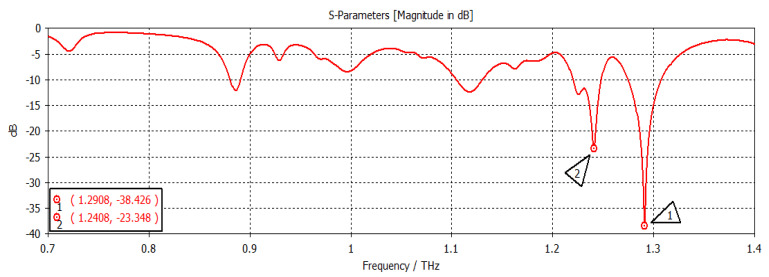
w_1_ = 150 µm and z_1_ = 100 µm.

The chemical potential was kept constant at 0.1 eV. The parameters w_1_, representing the width, and z_1_, representing the length of the graphene patch, were changed, and the results are shown in Figure 11. When w_1_ = 50 µm and z_1_ = 100 µm, as shown in Figure 11, the first and second frequencies were 1.2908 THz with a return loss of −46.9 dB and 1.2397 THz with a return loss of −31 dB, respectively. When the length was only changed to 300 µm, keeping w1 constant, as indicated in Figure 12, the frequency of operation changed to 1.2915 THz with a return loss of −43.327 dB, while the second frequency became 1.2416 THz with a return loss of −23.963 dB. When both w_1_ and z_1_ were changed to 100 µm and 300 µm, respectively (Figure 13), the first and second frequencies changed to 1.2929 THz and 1.2425 THz, respectively, while the return losses were −39.182 dB and −22.342 dB, respectively. Finally, in this experiment, the width and length were set at 150 µm and 100 µm, respectively, and the first and second frequencies, as indicated in Figure 14, changed to 1.2908 THz and 1.2408 THz, respectively. At the same time, the return losses were −38.426 dB and −23.348 dB, respectively.

From Table 3, where this work is compared with similar work using graphene as a tuning element, it can be seen that the filter operated at a higher frequency than filters and antennas designed using planar technology. The tunable bandwidth of the filter developed herewith, 1.289−1.297, was high, making it suitable for the tunability of various and broader application prospects.

## 6. Conclusions and Future Recommendation

In this research work, a second-order SIW filter was designed to operate at 1.2 THz. The magnetic iris of length d1, as shown in Figure 2, coupled two SIW resonators. For frequency tunability, graphene material was embedded into the substrate. The filter was made up of two layers of substrate, the first one being silicon, with a permittivity of 11.9, and the second substrate on top of the silicon substrate being silicon dioxide, with a permittivity of 3.9. The ground and top planes of the filter were gold plates. A thin graphene sheet was placed underneath the top gold plate to become sandwiched between the top gold plate and the silicon dioxide substrate. An external bias was applied between the top gold plate and the ground plate. This was to allow the chemical potential of graphene to be changed. As the chemical potential of graphene was changed, the center frequency of the designed filter changed at every instance.

The aspect ratio of the graphene sheet was also changed to change the frequency of the filter. As the width and the length of the graphene patch changed, the center frequency also changed; however, this method requires modifying the physical filter structure for a change in frequency, which may be undesirable, as compared with a change in frequency by simply changing an external voltage without changing the geometrics of the physical filter structure. More work in this research will be conducted on adding more layers of graphene sheets instead of one to improve the range in which the frequency can be tuned. Furthermore, the orientation of the graphene sheets will be interchanged between the horizontal and vertical positions.

## Figures and Tables

**Table 1:** SIW filter parameters.

Parameter	Dimension (µm)
h1	4
h2	4
x1	0.0034
y1	0.17
W	210
a1	50
d1	60
gap	10

**Table 2:** Graphene parameters.

Parameter	Value
Temperature	293 K
Relaxation time	0.1 ps
Thickness	0.34 nm

**Table 3:** Table of comparison with similar work.

Ref	Design Type	Technology	Center Frequency (THz)	Tunable Range (THz)	Material Used for Tuning
[15]	Band-stop filter	Planar microstrip	1	0.89–0.99	Graphene
[31]	Band-pass filter	Interdigital	1/1.5	1–1.03/1.5–1.57	Graphene
[33]	Band-stop filter	Planar microstrip	1.6	1.605–1.716	Graphene
[34]	Band-stop filter	Microstrip	0.2	0.2–0.245	Graphene
[14]	Antenna	Planar	0.65	0.65–0.7	Graphene
This work	Band-pass filter	SIW	1.2		Graphene

## Data Availability

Not applicable.
